# Eligibility of patients with advanced non-small cell lung cancer for phase III chemotherapy trials

**DOI:** 10.1186/1471-2407-9-130

**Published:** 2009-04-29

**Authors:** Janette Vardy, Ryan Dadasovich, Philip Beale, Michael Boyer, Stephen J Clarke

**Affiliations:** 1 Sydney Cancer Centre, Concord Repatriation General Hospital and Royal Prince Alfred Hospital, Sydney, NSW, Australia; 2 Sydney Cancer Centre, Concord Repatriation General Hospital, Hospital Rd, Concord, NSW, 2139 Australia; 3 Sydney Cancer Centre, Royal Prince Alfred Hospital, Missenden Rd, Camperdown, 2050, NSW, Australia

## Abstract

**Background:**

Evidence that chemotherapy improves survival and quality of life in patients with stage IIIB & IV non small cell lung cancer (NSCLC) is based on large randomized controlled trials. The purpose of this study was to determine eligibility of patients with advanced NSCLC for major chemotherapy trials.

**Methods:**

Physicians treating stage IIIB/IV NSCLC at Sydney Cancer Centre assessed patient eligibility for the E1594, SWOG9509 and TAX326 trials for patients presenting from October 2001 to December 2002. A review of the centre's registry was used to obtain missing data.

**Results:**

199 patients with advanced NSCLC were registered during the 14-month period. Characteristics of 100 patients were defined prospectively, 85 retrospectively: 77% males, median age 68 (range 32–88), 64% stage IV disease. Only 35% met trial eligibility for E1594 and 28% for SWOG9509 and TAX326. Common reasons for ineligibility were: co-morbidities 75(40%); ECOG Performance Status ≥2 72(39%); symptomatic brain metastasis 15(8%); and previous cancers 21(11%). Many patients were ineligible by more than one criterion.

**Conclusion:**

The majority of patients with advanced NSCLC were ineligible for the large chemotherapy trials. The applicability of trial results to advanced lung cancer populations may be limited. Future trials should be conducted in a more representative population.

## Background

There is level one evidence that patients with stage IIIB and IV non-small cell lung cancer (NSCLC) and good performance status benefit from chemotherapy, both in terms of survival and improved quality of life. A meta-analysis by the Non-Small Cell Lung Cancer Collaborative Group, comparing best supportive care (BSC) with BSC and chemotherapy reported a 27% reduction in the risk of death (p < 0.0001), with an increase in median survival of 1.5 months, in patients receiving cisplatin-based chemotherapy[1]. A phase III study randomising patients with stage IIIB (pleural effusion) or IV NSCLC to BSC or BSC and chemotherapy with mitomycin C, ifosfamide and cisplatin (MIC2), found a median survival of 6.7 months in the chemotherapy arm compared with 4.8 months in the BSC arm (p = 0.03)[2]. Quality of life improved from baseline to six weeks in the chemotherapy arm and deteriorated in the BSC arm (p = 0.007)[2]. A systematic review by Clegg found that the chemotherapeutic agents paclitaxel, docetaxel, gemicitabine and virorelbine improved survival in advanced NSCLC by 2–4 months compared to BSC, without compromising quality of life and that it was cost effective. [3]

Although the large chemotherapy versus BSC trials, and the more recent combination chemotherapy trials, reported a significant benefit from chemotherapy in both survival and quality of life in advanced NSCLC, all were performed in relatively young, highly selected groups of patients.

Some of the more influential recent studies performed for patients with NSCLC have included (i) European Co-operative Oncology Group (ECOG) E1594[4], (ii) South Western Oncology Group SWOG 9509[5] and (iii) TAX 326[6]. The ECOG 1594 trial compared paclitaxel and cisplatin with the following regimens: gemcitabine and cisplatin; docetaxel and cisplatin; or paclitaxel and carboplatin in 1155 patients. There was no difference in response rate (19%), median survival (7.9 months) or 1-year survival (33%) between the four arms. Time to progression was significantly better in the gemcitabine/cisplatin arm but there was greater renal toxicity. Initially patients with ECOG PS 2 were eligible, but the criteria were changed to exclude them after it became evident that patients with PS 2 had a significantly higher rate of adverse events than PS 0–1. Median survival in patients with PS 0 was 10.8 months, PS 1 7.1 months and in PS 2 3.9 months (p < 0.001)[4].

SWOG 9509 enrolled 406 patients with advanced NSCLC, all with ECOG performance status 0–1. Patients were randomised to receive either vinorelbine and cisplatin, or paclitaxel and carboplatin. There was no difference in median survival, 1 or 2 year survival or quality of life[5].

TAX326 randomised 1218 patients to receive either docetaxel and cisplatin, docetaxel and carboplatin or vinorelbine and cisplatin. Patients receiving docetaxel and cisplatin had a median survival of 11.3 months compared to 10.1 months for vinorelbine/cisplatin (p = 0.044), with no significant difference in median survival between vinorelbine/cisplatin and docetaxel/carboplatin, but poorer quality of life in the vinorelbine arm [6].

The highly selected patients treated in these clinical trials raises doubts about the applicability of their results to other patients with lung cancer. We therefore designed a questionnaire to determine how many of the total pool of patients with advanced NSCLC presenting to the Sydney Cancer Centre (excluding the Palliative Care department) would have been eligible for these recent randomised trials of chemotherapy and to document the reasons for ineligibility.

## Methods

Patients were recruited from the Royal Prince Alfred Hospital and Concord Repatriation General Hospital, Sydney, Australia from October 2001 until December 2002. Ethics committee approval was obtained from both participating hospitals. All physicians from Medical Oncology, Radiation Oncology, Respiratory Medicine and Thoracic Surgery were requested to complete a questionnaire at the time of the initial consultation for each new patient with Stage IIIB (pleural effusion) and Stage IV NSCLC presenting to their service. Basic demographic information was collected and histological subtype and staging was recorded as well as the presence (or not) of measurable or evaluable disease. Information on co-morbidities, prior history of malignancy and laboratory tests, including full blood count, liver function tests and creatinine, were recorded. Any previous treatment for lung cancer was documented and Eastern Co-operative Oncology Group (ECOG) performance status (PS) was evaluated. Physicians were asked to estimate patient's life expectancy as less than or greater than 12 weeks, to document weight loss of greater than 10% in the past 6 weeks, and to assess whether the patient was capable of giving informed consent. Patients seen by more than one service were only counted once.

All patients referred to the Sydney Cancer Centre are registered in a data base. This cancer registry was reviewed to determine how many patients with advanced NSCLC had been referred to the service during this period. A retrospective review of the medical records of patients not evaluated prospectively was undertaken to obtain as much information as possible: the hospital and oncology medical records were reviewed, the individual specialist was contacted and the hospital electronic pathology, laboratory and imaging results were examined.

Each patient's data were then reviewed to determine whether or not they met the eligibility criteria for each of the following trials: (i) ECOG E1594, (ii) SWOG 9509 and (iii) TAX 326.

### Statistical methods

Descriptive methods were used to summarise demographic characteristics. Analysis involved simple summary statistics, performed on Excel. Analysis of the prospective and retrospective data are presented separately as well as combined. Where information was unobtainable retrospectively (e.g. life expectancy) a conservative estimate was assumed. Where no evidence could be found that a patient had attended any appointment or had any investigations, it was assumed that they had been a non-attendee and they were excluded from the data set.

## Results

A total of 199 patients with advanced NSCLC were registered in the Sydney Cancer Centre data base between October 2001 and December 2002. Prospective questionnaires were obtained for 100 patients and data from retrospective chart review for an additional 85 patients. There were a further 14 patients who were registered but for whom no evidence could be found of their attending Sydney Cancer Centre or of having investigations during this period.

Patient characteristics are shown in table 1. The median age of the entire cohort was 68 years (age range 32–88 years) with 77% males and 23% females. Sixty-six patients (36%) had Stage IIIB disease, and 119 (64%) had stage IV disease. The most common histological diagnoses were: large cell 78 (42%), adenocarcinoma 67 (36%) and squamous cell carcinoma 23 (12%).

**Table 1:** Patient Characteristics

Patient Characteristics	Prospective Data n = 100 (%)	Retrospective Data n = 85 (%)	Total Cohort n = 185 (%)
Sex:			
Males	75 (75%)	68 (80%)	143 (77%)
Females	25 (25%)	17 (20%)	42 (23%)
Median age (years)	66(32–88)	68 (45–84)	68 (32–88)
Mean age	66	67	67
Staging:			
Stage IIIB (effusion)	37 (37%)	29 (34%)	66 (36%)
Stage IV	63 (63%)	56 (66%)	119 (64%)
Histological Subtype:			
Large cell carcinoma	50 (50%)	28 (33%)	78 (42%)
Adenocarcinoma	36 (36%)	31 (36%)	67 (36%)
Squamous cell carcinoma	10 (10%)	13 (15%)	23 (12%)
Bronchoalveolar	2 (2%)	4 (5%)	6 (3%)
Undifferentiated carcinoma	2 (2%)	4 (5%)	6 (3%)
Mixed	0 (0%)	2 (2%)	2 (1%)
Not available	0 (0%)	3 (3.5%)	3 (2%)
Measurable disease	88 (88%)	82 (96%)	170 (92%)
Evaluable disease	4 (4%)	2 (2%)	6 (3%)
Non-measurable disease	8 (8%)	1 (1%)	9 (5%)
ECOG Performance Status:			
ECOG 0	28 (28%)	17 (20%)	45 (24%)
ECOG 1	35 (35%)	26 (30.5%)	61 (33%)
ECOG 2	23 (23%)	15 (18%)	38 (20.5%)
ECOG 3 & 4	14 (14%)	20 (23%)	34 (19%)
Not available	0 (0%)	7 (8%)	7 (4%)
Estimated life expectancy:			
<12 weeks	34 (34%)	20 (23.5%)	54 (29%)
>12 weeks	66 (66%)	58 (68%)	124 (67%)
Not available	0 (0%)	7 (8%)	7 (4%)
Weight loss > 10% in 6 weeks	26 (26%)	26 (30.5%)	52 (28%)
Weight loss Not available	0 (0%)	10 (12%)	10 (5%)
Incapable of giving informed consent	1 (1%)	3 (3.5%)	4 (2%)

* retrospective patients were added to the Medical Oncology department referral base

# Blood abnormalities leading to exclusion from SWOG lung trial

Using the combined prospective and retrospective data set 64/185 (34%) patients were eligible for E1594, 53 (28%) for SWOG 9509 and 52 (28%) for TAX326: this included 3 patients for whom there were inadequate data to determine eligibility. (Table 2) Many patients were ineligible on more than one criterion. In the E1594 study 71 (38%) patients, and in the SWOG 9509 study 75 (40.5%), were ineligible on at least two criteria. (Table 3).

**Table 2:** Eligibility for E1594 and SWOG9509

	Prospective Data N = 100 (%)	Retrospective Data N = 85 (%)	Entire Cohort N = 185 (%)
E1594:			
Ineligible	61 (61%)	60 (70.5%)	121 (65%)
Eligible	39 (39%)	22 (26%)	61 (33%)
Not assessable	0 (0%)	3 (3.5%)	3 (2%)

SWOG 9509			
Ineligible	66 (66%)	66 (78%)	132 (71%)
Eligible	34 (34%)	16 (19%)	50 (27%)
Not assessable	0 (0%)	3 (3.5%)	3 (2%)

**Table 3:** Number (%) of Criteria Resulting in Ineligibility for Trials

No of Criteria	No of Patients E1594			No of Patients SWOG9509		
Resulting in Ineligibility	Prospective Data N = 100 (%)	Retrospective Data N = 85 (%)	Entire Cohort N = 185(%)	Prospective Data N = 100 (%)	Retrospective Data N = 85 (%)	Entire Cohort N = 185 (%)
1	32 (32%)	38 (45%)	71 (38%)	34 (34%)	41 (48%)	75 (40.5%)
2	22 (22%)	11 (13%)	33 (18%)	23 (23%)	12 (14%)	35 (19%)
3	6 (6%)	9 (11%)	15 (8%)	7 (7%)	11 (13%)	18 (10%)
4	1 (1%)	2 (2%)	3 (2%)	2 (2%)	2 (2%)	4 (2%)

* Eligibility for TAX326 is almost identical to SWOG9509 and so is not listed separately

The major reasons for ineligibility are outlined in Table 4. Seventy two (39%) were ineligible for all three trials due to a performance status of 2 or worse. This included 39 patients who were ECOG performance status 2; of whom only 15/39 (38%) met all other eligibility criteria for SWOG 9509 or TAX326.

**Table 4:** Number (%) of Ineligible Patients Based on Trials Exclusion Criteria*

	E1594			SWOG 9509		
	Prospective Data N = 100 (%)	Retro-spective Data N = 85 (%)	Entire Cohort N = 185 (%)	Prospective Data N = 100 (%)	Retro-spective Data N = 85 (%)	Entire Cohort N = 185 (%)
Performance Status	37 (37%)	35 (41%)	72 (39%)	37 (37%)	35 (41%)	72 (39%)

Co-morbidities	19 (19%)	31 (36%)	50 (27%)	34 (34%)	41 (48%)	75 (40%)

Previous History of Cancer	12 (12%)	9 (11%)	21 (11%)	12 (12%)	9 (10.5%)	21 (11%)

Brain Metastases	14 (14%)	1 (1%)	15 (8%)	14 (14%)	1 (1%)	15 (8%)

Non Evaluable or Non Measurable Disease	9 (9%)	1 (1%)	10 (5%)	9 (9%)	1 (1%)	10 (5%)

Abnormal Blood Parameter	2 (2%)	6 (7%)	8 (4%)	2 (2%)	7 (8%)	9 (5%)

Previous Chemotherapy	2 (2%)	10 (12%)	12 (6.5%)	2 (2%)	10 (12%)	12 (6.5%)

Unable to give Informed Consent	1 (1%)	3 (3.5%)	4 (2%)	1 (1%)	3 (3.5%)	4 (2%)

* Does not equal 100% due to many patients being excluded on more than one criterion – see Table 3

Seventy-five patients (40.5%) would have been excluded from SWOG 9509 and TAX326 for co-morbidities, most commonly severe or uncontrolled cardiac or pulmonary disease. The E1594 study has more liberal inclusion criteria and would have excluded 50 (27%) patients based on their co-morbidities. (Table 4)

Across all studies 21 (11%) patients were excluded due to second malignancies. Fifteen patients were not eligible because of unstable brain metastasis. Some of these patients might have subsequently qualified for inclusion in E1594 after cerebral radiotherapy.

Eleven patients would have been excluded from SWOG 9509 on the basis of abnormal blood test results: most commonly elevated creatinine and raised bilirubin. In 16 patients, blood results were not available, as they either hadn't been performed (generally because patients were too unwell to consider chemotherapy) or they were done in private laboratories and not available. For the purposes of determining eligibility these were regarded as being normal.

Ten patients were ineligible due to non-measurable disease and twelve patients because they had received prior chemotherapy.

## Discussion

The last decade has seen a shift from studies comparing BSC with chemotherapy in patients with advanced NSCLC, to studies comparing different chemotherapy combinations, administered either alone or in combination with molecular targeted agents, most commonly targeted against the epidermal growth factor receptor (EGFR) or vascular endothelial growth factor (VEGF). This has resulted in increasingly complex and expensive treatment regimens, and it is important that such therapies be evaluated in the target population. Consequently, we determined whether the patients enrolled in large chemotherapy trials were typical of the lung cancer patients presenting to cancer centres. Eligibility for the newer molecular targeted therapy trials were not included as these were not widely available at our centre at the time of this study; however a second study is in progress to assess this.

Of the 3 trials whose inclusion/exclusion criteria were evaluated, the ECOG-1594 study had the least restrictive criteria and would have excluded 121 (65%) of our NSCLC patients, whilst a further 3 had insufficient data available to determine their eligibility. Of the 61 (33%) that would have met E1594 eligibility criteria, 7 patients were estimated to have a life expectancy of less than 12 weeks, 4 had severe concomitant pulmonary disease and eight had weight loss of greater than 10% in the 6 weeks prior to assessment.

Life expectancy was estimated to be less than 12 weeks in 54 (29%) of patients. This is likely a conservative estimate, as physicians are known to consistently overestimate life expectancy in people with advanced cancer[7], and where life expectancy was not documented we assumed it was greater than 12 weeks. Physicians estimated a life expectancy of <12 weeks in 6 patients who met all eligibility criteria for E1594, SWOG 9509 and TAX 326.

Weight loss is a poor prognostic factor for patients with advanced NSCLC[8,9] Fifty two (28%) patients had documented weight loss of greater than 10% within the preceding six weeks: of these patients 12 (6%) were eligible for the E1594 trial and 13 (7%) met eligibility criteria for both the SWOG 9509 and TAX 326 trials.

Excluding patients with ECOG PS of 2–4 immediately excluded 72 (39%) of our patients with advanced NSCLC. Of those with a performance status of 2, only 15/39 (38%) would have met all the other eligibility criteria for SWOG 9509 or TAX326.

Co-morbidities were a major cause of trial ineligibility: 40.5% in SWOG 9509 and 27% in E1594. With approximately 86% of lung cancers in men and 49% in women being current smokers, or having a significant past history of smoking[10], it is not surprising that there is a high rate of co-morbidities, particularly pulmonary (e.g. chronic obstructive pulmonary disease) and cardiovascular disease. In addition 11% of our patients had a past history of a previous malignancy, excluding non-melanoma skin lesions and cervical cancer in situ. A total of 35 (19%) patients were excluded for having non-measurable disease, previous malignancy and/or prior receipt of chemotherapy. Of these, 24 (69%) remained ineligible on other grounds, and 11 patients were rendered ineligible on these criteria only, and would not necessarily have been restricted from receiving chemotherapy off trial.

The major limitation of this study is that it is not population based. Due to failure of physicians to complete the questionnaire on every new patient presenting to the Sydney Cancer Centre with advanced NSCLC, we were not able to prospectively survey consecutive patients. Examination of our lung cancer data base reveals that it is likely that our study obtained data on 93% of patients who had presented to the Sydney Cancer Centre with advanced NSCLC during the study period; although almost half of the data were obtained retrospectively. However, our study likely underestimates the number of patients that would be excluded from advanced NSCLC trials, as the sickest patients and patients with other co-morbidities are often not referred to a Cancer Centre, but instead are managed by their family physician, geriatrician or palliative care services with symptomatic treatment only. It is likely that a true population based study would result in an even smaller percent of patients meeting inclusion criteria.

Whilst cisplatin-based chemotherapy regimens have been shown to increase survival and quality of life in patients who are eligible for large clinical trials compared to those treated with best supportive care, we need to recognise limitations in generalisability of results of those trials to the broader population with advanced lung cancer. The median age of our cohort was 68 years, consistent with the median age of presentation of patients with advanced NSCLC, whereas the median age in E1594, SWOG9509 and TAX326 was 61–63 years. It is only recently that we have seen the emergence of studies investigating the effects of chemotherapy in the elderly[11,12], as well as ongoing studies in those with poorer performance status.

## Conclusion

Our study demonstrates that 65–71% of ANSCLC patients presenting to Sydney Cancer Centre, would not have been eligible for the major lung cancer trials E1594, SWOG 9509 and TAX356. There is a lack of evidence-based data on which to base treatment decisions in the majority of patients with advanced NSCLC. The optimal treatment for these patients is not known.

Our recommendations are that future trials need to be developed with more liberal eligibility and/or to target specific subsets of patients within the population with advanced NSCLC to ensure that results are applicable to more patients.

## Competing interests

The authors declare that they have no competing interests.

## Authors' contributions

JV and SJC were responsible for concept and design, acquisition of data, analysis and interpretation of data and writing of the manuscript. RD contributed to acquisition of data. PB and MB contributed to study design and patient recruitment. All authors reviewed the manuscript.

## Pre-publication history

The pre-publication history for this paper can be accessed here:

http://www.biomedcentral.com/1471-2407/9/130/prepub
